# Pharmacological inhibition of sodium-calcium exchange activates NADPH oxidase and induces infection-independent NETotic cell death

**DOI:** 10.1016/j.redox.2021.101983

**Published:** 2021-04-26

**Authors:** Minoru Inoue, Masahiro Enomoto, Michio Yoshimura, Takashi Mizowaki

**Affiliations:** a Department of Radiation Oncology and Image-applied Therapy, Kyoto University Graduate School of Medicine, Kyoto, Japan; b Princess Margaret Cancer Centre, Department of Medical Biophysics, University Health Network, University of Toronto, Toronto, Ontario, Canada

**Keywords:** Neutrophil, Neutrophil extracellular traps, Amiloride, Sodium-calcium exchanger, NADPH oxidase, ROS, NETs, neutrophil extracellular traps, EIPA, 5-(N-ethyl-N-isopropyl)amiloride, MIA, 5-(N-Methyl-N-isobutyl)amiloride, DMA, 5-(N,N-Dimethyl)amiloride hydrochloride, ROS, reactive oxygen species, NCX, sodium-calcium exchanger, NHE, sodium-hydrogen exchanger, NADPH, dihydronicotinamide-adenine dinucleotide phosphate, DPI, diphenyleneiodonium chloride, BSA, Bovine serum albumin, NAC, *N*-acetylcysteine, MSU, monosodium Urate, ELISA, Enzyme-linked immuno-sorbent assay

## Abstract

In addition to its function of innate immunity against invading pathogens, neutrophil extracellular traps (NETs) promote thrombosis, autoimmune disease, and cancer metastasis; therefore, unnecessary exposure to the triggers of infection-independent NET generation should be avoided. We herein show that inhibition of forward-mode Na^+^/Ca^2+^ exchange by amiloride analogs, 5-(N-ethyl-N-isopropyl)amiloride (EIPA) and 5-(N-Methyl-N-isobutyl)amiloride (MIA), triggers NETotic cell death independently of infectious stimuli. Isolated human neutrophils treated with EIPA and MIA undergo NETotic cell death by an increase of intracellular Ca^2+^ following activation of NADPH oxidase and the resultant upregulation of intracellular ROS. EIPA- and MIA-mediated intracellular Ca^2+^ increase is attributed to the competitive binding of EIPA and MIA against Na^+^ to Na^+^/Ca^2+^ exchanger 1 (NCX1). These results demonstrate a new mechanism of infection-independent NET generation and implicate NCX1 as a physiologic regulator of intracellular calcium balance and NETotic cell death.

## Highlights

• Two of the amiloride analogs, EIPA and MIA, induce NETotic cell death without infectious stimuli.
• EIPA and MIA inhibit the forward-mode Na^+^/Ca^2+^ exchange and promote the intracellular Ca^2+^ overload.
• Intracellular Ca^2+^ overload by EIPA and MIA activates NADPH oxidase, elevates intracellular ROS level, and induces resultant NETotic cell death.

## Graphical abstract

**Figure:**
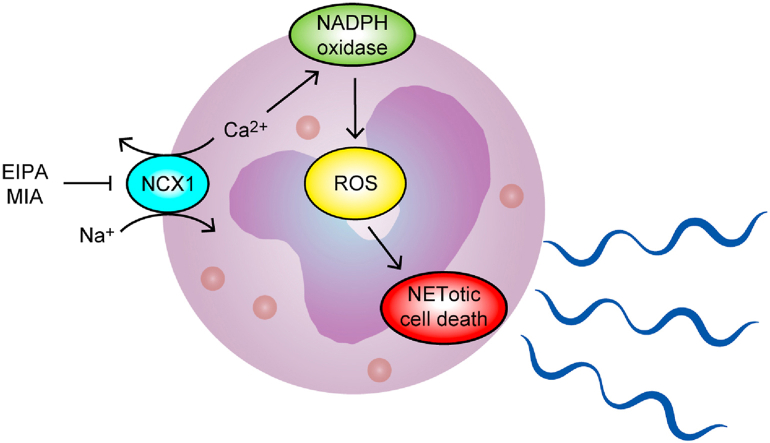

## 1 Introduction

Neutrophils release their own nuclear and mitochondrial DNA decorated with cytosolic and granule proteins, known as neutrophil extracellular traps (NETs) [1,2]. NETs are generated over several hours accompanied by cell death (i.e., NETotic cell death) [3] or within minutes without cell death (i.e., non-lytic NET formation) [4,5], and entrap and kill various pathogens to prevent their dissemination [1,4,5]. In addition to this function, there is emerging evidence that NETs are associated with the promotion of thrombosis [6], autoimmune disease [7,8], and cancer metastasis [[9], [10], [11]]. Therefore, understanding the triggers for NET release, such as the iatrogenic triggers, may be an important medical issue for preventing unexpected side effects. We previously reported that accumulation of oxidized serum albumin triggers infection-independent NETotic cell death by intracellular accumulation of reactive oxygen species (ROS), which contributes to the promotion of pulmonary cancer metastasis [11].

Amiloride and its derivatives, known as potassium-sparing diuretic agents, have been found to inhibit the activitiy of forward-mode Na^+^/Ca^2+^ exchanger (NCX) in the cardiac sarcolemmal membrane [12,13] and Na^+^/H^+^ exchanger (NHE) in Malpighian tubules [14]. The inhibition of the forward-mode activity of NCX may promote the accumulation of intracellular Ca^2+^ by suppressing Ca^2+^ efflux and Na^+^ influx, which, in turn, activates nicotinamide adenine dinucleotide phosphate (NADPH) oxidase and produces superoxide [15]. Inhibiting the activity of NHE may predispose cells to starvation of serum albumin by inhibition of macropinocytosis [16]. As serum albumin has a free thiol group at the cysteine 34 position [17], inhibition of NHE may cause a lack of intracellular free thiols. These mechanisms may hypothetically contribute to the accumulation of intracellular ROS and resultant NET release. To date, the influence of amiloride and its derivatives on NET release remains elusive.

In this study, we demonstrate that inhibition of the forward-mode Na^+^/Ca^2+^ exchange by amiloride derivatives, 5-(N-ethyl-N-isopropyl)amiloride (EIPA) and 5-(N-Methyl-N-isobutyl)amiloride (MIA) triggers infection-independent NETotic cell death while the activity of NHE is not involved in EIPA- and MIA-mediated NETotic cell death. Our findings identify a previously unknown mechanism in NET release and provide further insight to the Na^+^/Ca^2+^ exchange in the regulation of intracellular calcium balance and NETotic cell death.

## 2 Materials and methods

### 2.1 Neutrophil isolation

Human whole blood from healthy volunteers was collected into EDTA-2K containing tube. Neutrophils were isolated by Polymorphprep (Alere Technologies, Germany) according to the manufacturer's instructions. Neutrophils were re-suspended in Roswell Park Memorial Institute (RPMI) 1640 (Gibco/Life Technologies, MA, USA) without phenol red supplemented with 1% fetal bovine serum (FBS; Gibco/Life Technologies, MA, USA). Neutrophil purity was established to be routinely >90%, as assessed by May-Grünwald Giemsa (Sigma-Aldrich, MO, USA) staining.

### 2.2 Cell culture and reagents

Isolated neutrophils were cultured in RPMI-1640 medium supplemented with 1% FBS in uncoated polystyrene plates. All cultures were maintained in a well-humidified incubator with 5% CO_2_ and 95% air at 37 °C. Amiloride hydrochloride hydrate, 5-(N-Ethyl-N-isopropyl)amiloride (EIPA), 5-(N-Methyl-N-isobutyl)amiloride (MIA), 5-(N,N-Dimethyl)amiloride hydrochloride (DMA), phenamil hydrochloride, and benzamil methanesulfonate salt (Sigma-Aldrich, MO, USA) were dissolved in dimethyl sulfoxide (DMSO). Ionomycin (Nacalai Tesque, Japan), BAPTA-AM (Sigma-Aldrich, MO, USA), diphenyleneiodonium chloride (DPI; Sigma-Aldrich, MO, USA), and SEA0400 (Sigma-Aldrich, MO, USA) were dissolved in DMSO. Bovine serum albumin (BSA; BioShop Canada Inc., Canada), Monosodium Urate (MSU; InvivoGen, CA, USA) and *N*-acetylcysteine (NAC; Sigma-Aldrich, MO, USA) were dissolved in phosphate buffered saline (PBS).

### 2.3 Albumin depletion from serum

Bovine serum albumin (BSA) was depleted from serum using polystyrene columns (ThermoFisher Scientific, MA, USA) packed with albumin-binding resin (CaptureSelect MultiSpecies Albumin Depletion; ThermoFisher Scientific, MA, USA). Five hundred microliters of serum was loaded into the column packed with 2.5 mL of resin and washed using PBS by gravity flow. These processes were repeated 3–5 times until the concentration of albumin was less than 0.2 g/dL. Flow-through fractions were collected and immediately passed through a 0.22 μm sterile filter (MilliporeSigma, MA, USA) to remove bacterial contamination. The degree of albumin depletion was confirmed by quantifying the concentrations of total protein and albumin using the Bradford protein assay (Bradford Reagent, Sigma-Aldrich, MO, USA) and bromocresol green assay (BCG albumin assay kit, Sigma-Aldrich, MO, USA), respectively.

### 2.4 Imaging of NETs

Human neutrophils were cultured under the designated condition for 5 h. Hoechst33342 (2 drops/mL; NucBlue Live Cell ReadyProbes Reagent, ThermoFisher Scientific, MA, USA) and SytoxOrange (1:1000; Invitrogen Inc., CA, USA) were added to the culture medium. Neutrophils were then observed using the EVOS FL Cell Imaging System (Life technologies, CA, USA).

### 2.5 Immunocytochemistry

Human neutrophils cultured under the designated condition for 5 h were fixed with 4% paraformaldehyde (PFA) for 10 min and permeabilized with PBS containing 0.2% Tween20 for 10 min. After washing with PBS three times and incubating for 20 min in the SuperBlock (PBS) Blocking Buffer (ThermoFisher Scientific, MA, USA), cells were labeled with a rabbit anti-citrullinated histone H3 antibody (1:1000; ab5103, Abcam, MA, USA) for 1 h at room temperature. After washing with PBS containing 0.1% Tween20 three times, citrullinated histone H3 was labeled with Alexa Fluor 488 anti-rabbit IgG secondary antibody (1:1000; Invitrogen Inc., CA, USA) for 1 h at room temperature. After an additional 3 washes in PBS containing 0.1% Tween20, DNA was stained with DAPI (NucBlue Fixed Cell ReadyProbes Reagent, ThermoFisher Scientific, MA, USA). Slides were mounted in ProLong Gold anti-fade reagent (ThermoFisher Scientific, MA, USA). Images were acquired using the EVOS FL Cell Imaging System (Life technologies, CA, USA).

### 2.6 Quantification of NETs within cell culture supernatant

Human neutrophils (5 × 10^5^) were cultured in 24-well culture plates under the designated condition. After 5-h incubation, the culture medium was collected after vigorous agitation. The medium was centrifuged at 400×*g* for 5 min and the supernatant was used for the quantification of NETs. We utilized a previously established ELISA assay for the detection of elastase-DNA complexes [24] with slight modifications. A rabbit polyclonal anti-neutrophil elastase antibody (1:500; ab131260, Abcam, MA, USA) in 100 mM of bicarbonate/carbonate coating buffer (50 μL in total) was coated onto 96-well microtiter plates (FluoroNunc/LumiNunc white Maxisorp, ThermoFisher Scientific, MA, USA) overnight at 4 °C. After blocking in 2% BSA at 37 °C for 2 h, the mixture of 50 μL of cell culture supernatant was loaded per well and incubated at room temperature for 1 h. After washing three times with PBS containing 0.05% Tween 20 and 5 mM EDTA (PBST-EDTA), a horseradish peroxidase conjugated anti-DNA antibody (1:100; D5425–3–100, Zymo Research Corporation, CA, USA) in PBST-EDTA (50 μL in total) was added to each well and incubated at room temperature for 1 h. After washing three times with PBST-EDTA, the peroxidase substrate (Glo Substrate Reagent Pack (DY993), Bio-Techne Corporation/R&D Systems, MN, USA) was added. The intensity of chemiluminescence was measured using a plate reader (Multilabel Plate Reader ARVO X5 [VICTOR X5], PerkinElmer, MA, USA). For analysis, the luminescence of the blank well was subtracted from the luminescence of each sample.

### 2.7 Quantification of extracellular DNA

Human neutrophils (5 × 10^5^) were cultured in 24-well culture plates under the designated condition. The medium was centrifuged at 400×*g* for 5 min and the supernatant was obtained. The concentrations of total extracellular DNA in the cell culture supernatant were quantified by Qubit 3 fluorometer (ThermoFisher Scientific, MA, USA) and dsDNA HS assay kit (ThermoFisher Scientific, MA, USA). The reaction mixture for each sample consisted of 20 μL of the culture medium and 180 μL of working solution (dsDNA HS reagent:dsDNA HS buffer = 1:200) was prepared in Qubit Assay Tubes (ThermoFisher Scientific, MA, USA).

### 2.8 Intracellular ROS detection

Intracellular ROS level was measured using the DCFDA Cellular ROS detection Assay Kit (Abcam, MA, USA) according to the manufacturer's protocol. Isolated human neutrophils were treated with 20 μM DCFDA for 30 min. After centrifugation (400×*g* for 5 min), neutrophils were re-suspended in the culture medium and seeded onto 96-well plates at 1 × 10^5^ cells per well. The ROS level represented by the fluorescence intensity of DCFDA was quantified using a fluorescence plate reader (TECAN Infinite M200Pro, Tecan Group Ltd., Switzerland).

### 2.9 Imaging of albumin internalized by neutrophils

Human neutrophils (5 × 10^5^) were cultured on glass coverslips in 24-well culture plates with EIPA or MIA for 10 min. For visualization of the internalization of supplemented BSA by neutrophils, PBS solution of albumin-fluorescein isothiocyanate conjugate (Sigma-Aldrich, MO, USA) was added to the culture medium at a final concentration of 0.02 g/dL. After washing three times with PBS, neutrophils were fixed with 4% PFA for 10 min. After an additional washing three times with PBS, DNA was stained with DAPI (NucBlue Fixed Cell ReadyProbes Reagent, ThermoFisher Scientific, MA, USA). Slides were mounted in ProLong Gold anti-fade reagent (ThermoFisher Scientific, MA, USA). Images were acquired using a Leica SP8 confocal microscope (Leica microsystems, Germany).

### 2.10 Quantification of NADPH and NADP^+^

Human neutrophils (3 × 10^6^) were treated with DMSO, EIPA, and MIA with or without BAPTA-AM. After 60-min incubation, cells were harvested and subjected to quantify the ratio of NADP^+^/NADPH ratio by NADP/NADPH Assay Kit (Dojindo, Japan) following the manufacture's protocol. The ratio of NADP^+^/NADPH in each treatment group was calculated.

### 2.11 Fluorescent analysis of intracellular Ca^2+^ and pH

Intracellular Ca^2+^ and pH were measured by Fluo-4 AM (ThermoFisher Scientific, MA, USA) and pHrodo Red (ThermoFisher Scientific, MA, USA) according to the manufacturer's protocols. Human neutrophils were treated with 5 μM Fluo-4 AM and 5 μM pHrodo Red for 15 min. After centrifugation (400×*g* for 5 min), neutrophils were re-suspended in the culture medium with or without drugs as indicated and then seeded onto 96-well plates at 1 × 10^5^ cells per well. The fluorescence intensity of Fluo-4 and pHrodo Red were quantified using a fluorescence plate reader (TECAN Infinite M200Pro, Tecan Group Ltd., Switzerland).

### 2.12 Simultaneous imaging of intracellular Ca^2+^ and Na^+^

Isolated human neutrophils were treated with 4.5 μM Fura Red AM (ThermoFisher Scientific, MA, USA) and 9.5 μM CoroNa Green AM (ThermoFisher Scientific, MA, USA) for 20 min. After washing two times with PBS, neutrophils were re-suspended in RPMI-1640 with 1% FBS and seeded onto 35-mm glass bottom dishes at 3 × 10^5^ cells per well. Cells were observed using FLUOVIEW FV10i (Olympus, Tokyo, Japan) every 20 s for 10 min. In order to perform ratiometric measurements, Fura Red AM was excited at 405 nm and 559 nm. The injection of EIPA and MIA into the culture dishes was gently performed using a syringe with a flexible tube attached to the lid of culture dishes so as not to interrupt the process of image acquisition. Time to time fluorescent intensity of Fura Red AM and CoroNa Green AM of the cells was quantified by MetaMorph software (Molecular Devices, Inc; USA).

### 2.13 Ligand docking analysis

The computational ligand docking analysis was conducted using Rosetta Ligand Docking Protocol on the ROSIE sever (https://rosie.graylab.jhu.edu/) [[18], [19], [20], [21]]. The ligand SDF files were downloaded from PubChem (https://pubchem.ncbi.nlm.nih.gov/). A homology model of human NCX1 was built using Rosetta Web Server [22]. In the amino acid sequence of human NCX1 (UniProtKB accession number: P32418), the large f-loop which comprehends two regulatory Ca^2+^-binding domains (CBD1 and CBD2 [residues 253–800]) [23] was deleted, and then the sequence was threaded onto the X-ray crystal structure of *Methanocaldococcus jannaschii* NCX (PDB 3V5U). The [x, y, z] coordinates of starting position were approximated by Na ^+^ binding sites of *Methanocaldococcus jannaschii* NCX.

### 2.14 Statistical analyses

Data are presented as mean and standard deviation (s.d.). The significance of difference between two independent subjects and among multiple subjects was determined using the Student's *t*-test and Dunnett's test, respectively. Data were obtained from at three independent experiments with 1–3 technical replicates. All tests were two-tailed with a *P* value < 0.05 considered to be significant. All calculations and analyses were performed in R (version 4.0.2; http://www.R-project.org).

## 3 Results

### 3.1 The amiloride derivatives EIPA and MIA trigger NETotic cell death following the upregulation of intracellular ROS

**Fig. 1:**
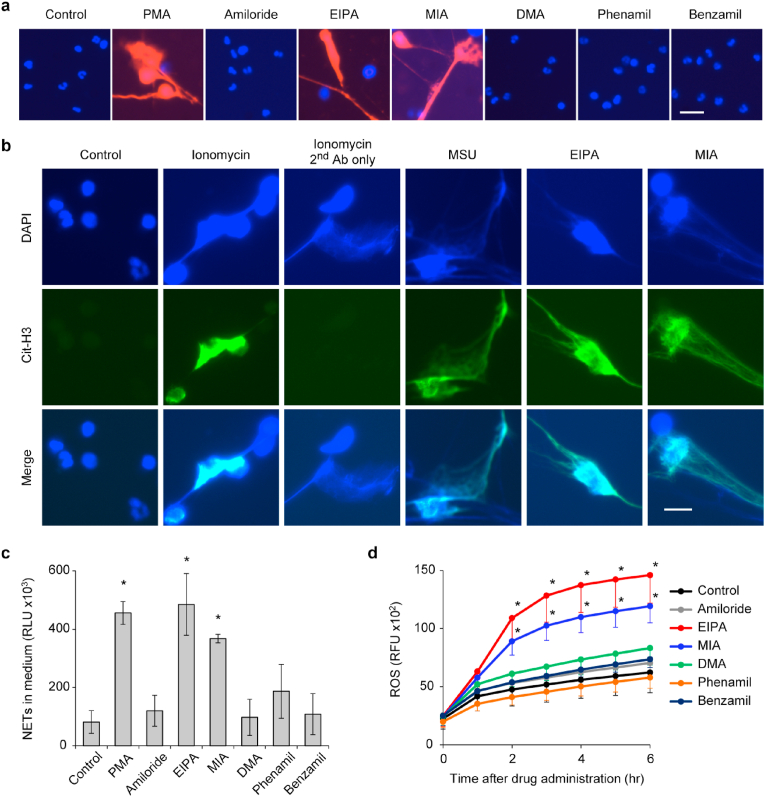
**| Amiloride derivatives EIPA and MIA trigger NETotic cell death following the upregulation of intracellular ROS.** (a–c) Human neutrophils were treated with the indicated compounds (final concentration: 75 μM for all amiloride compounds, 20 nM for PMA, and 200 μg/mL monosodium urate [MSU]) for 5 h. (a) Representative images of neutrophils stained with cell-permeable DNA dye, Hoechst 33342 (blue), and cell-impermeable DNA dye, SytoxOrange (red). Bar = 20 μm. (b) Representative immunofluorescence images are shown of neutrophils stained with DAPI (blue) and anti-citrullinated histone H3 (Cit-H3, green). Bar = 10 μm. (c) Levels of NETs within cell culture supernatant derived from neutrophils with each treatment. (d) Intracellular ROS levels within human neutrophils treated with amiloride and its analogs. (c, d) Results represent the mean ± s.d. (*n* = 3; biological replicates, significant differences were compared with the control at *P < 0.05 by Dunnett's test). (For interpretation of the references to colour in this figure legend, the reader is referred to the Web version of this article.)

Table S1 shows the amiloride analogs assessed in this study: 5-amino-substituted analogs (EIPA, MIA, and DMA), and 2-guadino-substituted analogs (phenamil and benzamil). Under the culture condition of isolated human neutrophils, we examined whether amiloride and its analogs trigger NET release. Neutrophils treated with EIPA and MIA for 2 h showed a similar morphological change to that observed in those treated with phorbol 12-myristate 13-acetate (PMA; Fig. S1). In contrast, neutrophils treated with the other amiloride compounds maintained a round or spherical shape similar to vehicle-treated neutrophils (Fig. S1). A 5-h incubation under the treatment of EIPA and MIA led neutrophils to NETotic cell death, characterized by elongated DNA fibers (Fig. 1a and S2) in association with the NET marker citrullinated histone H3 [25], which is identical to NETs triggered by the calcium ionophore ionomycin [25] and monosodium urate crystals (MSU) [26] (Fig. 1b). In contrast, the neutrophils treated with the other compounds and the vehicle-treated control remained alive (Fig. 1a). Consistent with this result, the levels of NETs in the cell culture supernatant were elevated under EIPA and MIA treatment conditions, but not other treatment conditions (Fig. 1c). Higher concentrations of amiloride and DMA did not exhibit cytotoxicity, whereas higher concentrations of phenamil and benzamil induced a modest cytotoxicity and sporadic formation of NETs (Figs. S3a and b). Collectively, only EIPA and MIA efficiently trigger NETotic cell death.

Subsequently, we assessed the intracellular levels of ROS under the treatment conditions of amiloride and its analogs because upregulation of intracellular ROS is a key process in the initiation of NETotic cell death [3]. After a 2-h incubation, intracellular ROS was significantly elevated in the neutrophils treated with only EIPA and MIA (Fig. 1d), suggesting that EIPA and MIA induce NETotic cell death by a mechanism involving the upregulation of intracellular ROS.

### 3.2 EIPA and MIA develop intracellular calcium overload and accumulation of NADPH oxidase-derived ROS in neutrophils

**Fig. 2:**
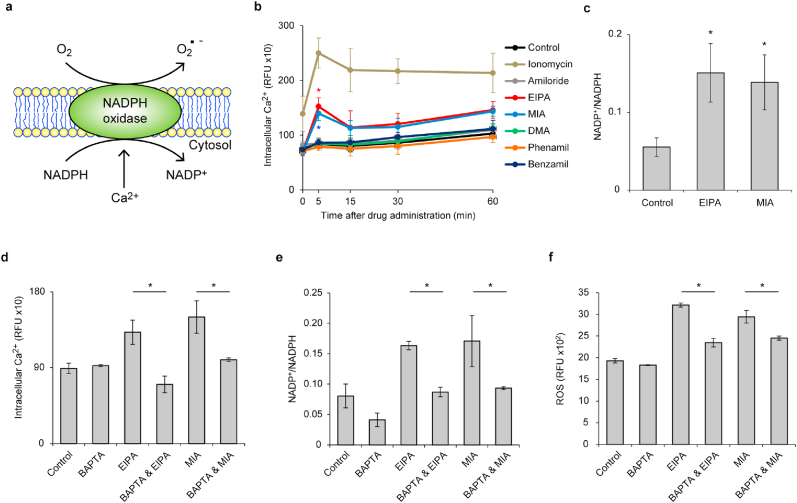
**| EIPA and MIA promote intracellular calcium overload and accumulation of NADPH oxidase-derived ROS in human neutrophils.** (a) Schematic representation of NADPH oxidase-dependent ROS generation. (b–f) Human neutrophils were treated with the indicated compounds (final concentration: 75 μM, 1 μM, and 8 μM for amiloride compounds, ionomycin, and BAPTA-AM, respectively) for 5 min (d), 60 min (c, e), or 2 h (f). (b, d) Intracellular Ca^2+^ levels in each treatment group were measured by the fluorescent intensity of Fluo-4 AM. (c, e) The ratio of NADP^+^/NADPH in each treatment group was calculated by the concentrations of NADP^+^ and NADPH. (f) Intracellular ROS level in each treatment group was measured by DCFDA fluorescence. (b–f) Results represent the mean ± s.d. (*n* = 3; biological replicates; Significant differences were compared with the control at *P < 0.05 by Dunnett's test (b, c); *P < 0.05 by Student's *t*-test (d, f); (b) the red asterisks represent statistical significance between the control and EIPA-treated groups; the blue asterisks represent statistical significance between the control and MIA-treated groups). (For interpretation of the references to colour in this figure legend, the reader is referred to the Web version of this article.)

In order to elucidate the mechanism by which EIPA and MIA upregulate ROS within neutrophils, we investigated the possibility of Ca^2+^-dependent activation of NADPH oxidase being involved in EIPA- and MIA-induced ROS increase (Fig. 2a). For this purpose, we assessed whether EIPA or MIA had an effect on the changes of intracellular Ca^2+^ within the neutrophil. Quantitative analysis of the fluorescence signal of the calcium indicator Fluo-4 AM demonstrated that intracellular Ca^2+^ rose within 5 min after the addition of EIPA and MIA as well as ionomycin, a calcium ionophore (Fig. 2b). This effect was not observed in the neutrophils treated with the other amiloride compounds even during experiments that included a longer observation period (Fig. 2b). Following this result, the ratio of NADP^+^ to NADPH was elevated in the neutrophils treated with EIPA and MIA compared to the control (Fig. 2c), suggesting that NADPH oxidase was activated by EIPA and MIA.

To further demonstrate the causal link between intracellular Ca^2+^ and NADPH oxidase activity, we used a cell-permeable Ca^2+^ chelator, BAPTA-AM. The addition of BAPTA-AM abrogated the increase of the intracellular Ca^2+^ provided by EIPA and MIA (Fig. 2d), and mitigated the EIPA- and MIA-mediated increase of the NADP^+^/NADPH ratio (Fig. 2e). Consistent with these results, the accumulation of intracellular ROS observed in EIPA- and MIA-treated neutrophils was inhibited by the addition of BAPTA-AM (Fig. 2f). Taken together, Ca^2+^-dependent activation of NADPH oxidase causes the intracellular accumulation of ROS by EIPA and MIA.

### 3.3 EIPA and MIA block the forward mode activity of NCX by competitive binding against sodium ions in neutrophils

**Fig. 3:**
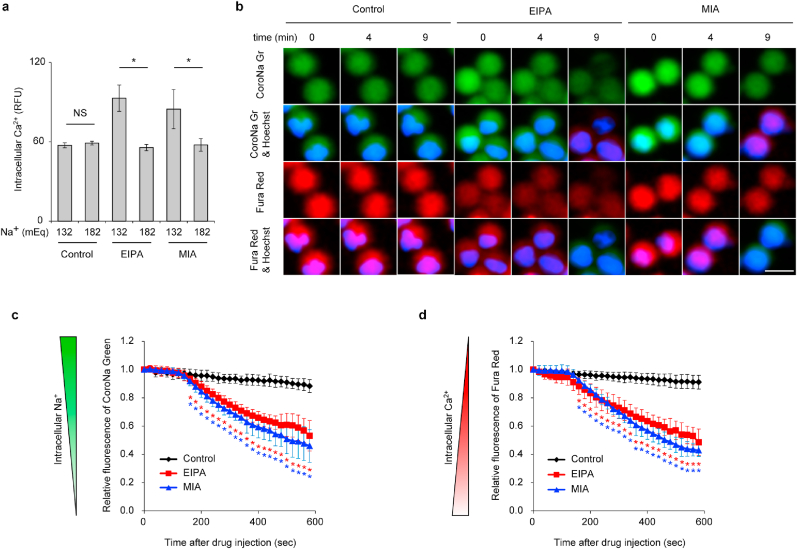
**| EIPA and MIA block the forward mode activity of NCX by competitive binding against sodium ion in human neutrophils.** (a) Human neutrophils cultured in the original medium (i.e, 132 mEq of Na^+^) or the medium containing 182 mEq of Na^+^ were treated with 75 μM of EIPA and MIA. Intracellular Ca^2+^ level in each group was measured by the fluorescent intensity of Fluo-4 AM. (b, c, d) Human neutrophils loaded with Fura Red AM and CoroNa Green AM were subjected to the time-lapse imaging. Seventy-five micro-molar of EIPA and MIA were added to the culture medium at time 0. (b) Representative images are shown. Bar = 10 μm. (c, d) Changes in the intracellular Ca^2+^ and Na^+^ after administration of EIPA and MIA. Intracellular Ca^2+^ and Na^+^ were analyzed using the fluorescent intensity of Fura Red AM and CoroNa Green AM, respectively. Each fluorescent intensity was normalized by the one measured at time 0. (a, c, d) Results represent the mean ± s.d. (*n* = 3 (a); *n* = 20 cells (c, d), *P < 0.05 and NS = not significant by Student's *t*-test. The red asterisks represent statistical significance between the control and EIPA-treated groups in each time point. The blue asterisks represent statistical significance between the control and MIA-treated groups (c, d). (For interpretation of the references to colour in this figure legend, the reader is referred to the Web version of this article.)

Next, we investigated the underlying mechanism by which EIPA and MIA promote the overload of Ca^2+^ in neutrophils. Considering the inhibitory effect of amiloride and its derivatives against the forward mode activity of NCX (i.e., Ca^2+^ efflux and Na^+^ influx) [27,28] and the specific expression of NCX1 in human neutrophils (Human Protein Atlas, https://www.proteinatlas.org/ENSG00000183023-SLC8A1/blood) compared to the other isoforms (i.e., NCX2 [https://www.proteinatlas.org/ENSG00000118160-SLC8A2/blood] and NCX3 [https://www.proteinatlas.org/ENSG00000100678-SLC8A3/blood]), we analyzed the binding ability of amiloride and its derivatives to NCX1 using *in silico* docking calculations. The long alkyl group on the 5-amino nitrogen atom in EIPA and MIA contributed to stronger hydrophobic interaction with transmembrane helices constituting Na^+^ binding sites (Fig. S4). These effects led to the conformation changes of the helices accompanied by hydrogen bonding between S145 and E148, resulting in the disruption of Na^+^ binding to these helices (Figs. S4b and c). We further examined whether the addition of Na^+^ to the culture medium prevents the increase of intracellular Ca^2+^ by EIPA and MIA. The fluorescence signal for intracellular Ca^2+^ under the existence of EIPA and MIA was dramatically reduced in the culture medium containing higher Na^+^ (Fig. 3a). These results suggest that EIPA and MIA possess stronger binding ability to the transmembrane helices constituting the Na^+^ binding sites in NCX1 than amiloride and the other amiloride derivatives.

To further provide evidence that EIPA and MIA block the forward mode activity of NCX in neutrophils, we analyzed the changes in intracellular Ca^2+^ and Na^+^ with and without EIPA and MIA. For this purpose, neutrophils were loaded with Fura Red AM and CoroNa Green AM for visualizing intracellular Ca^2+^ and Na^+^, respectively. Time-lapse imaging of these two dye-loaded neutrophils demonstrated that the increase of intracellular Ca^2+^ and the decrease of intracellular Na^+^ simultaneously occurred 3 min after the addition of EIPA and MIA (Fig. 3b–d, Videos S1 and S2) while these changes were not observed in vehicle-treated neutrophils (Fig. 3b–d, Video S3). Ionomycin-treated neutrophils exhibited the increase of intracellular Ca^2+^ but no significant changes were observed in intracellular Na^+^ (Fig. S5, Video S4), which is consistent with the known mechanism by which ionomycin forms an electrically neutral complex with Ca^2+^ and promotes the diffusion of Ca^2+^ through the plasma membranes [29].

In order to additionally investigate whether the activity of reverse mode NCX (i.e., Ca^2+^ influx and Na^+^ efflux) is involved in EIPA- and MIA-mediated NET release, we used an inhibitor of the reverse mode activity of NCX, SEA0400 [30]. Neutrophils treated with SEA0400 with or without EIPA and MIA showed no significant reduction in intracellular Ca^2+^ levels (Fig. S6a). Consistent with this result, the SEA0400 treatment did not prevent EIPA- or MIA-mediated NETotic cell death (Fig. S6b). Since reverse mode NCX activity is elicited in cardiomyocytes under pathophysiological conditions, such as ischemia/reperfusion injury [31] and heart failure [32], the activity of reverse mode NCX may be suppressed in neutrophils, and, thus, reverse mode NCX may not be involved in EIPA- and MIA-mediated NETotic cell death. Taken together, these results indicate that EIPA and MIA cause intracellular Ca^2+^ overload by the inhibition of the forward mode of activity of NCX.

### 3.4 EIPA and MIA do not inhibit the uptake of serum albumin by neutrophils

**Fig. 4:**
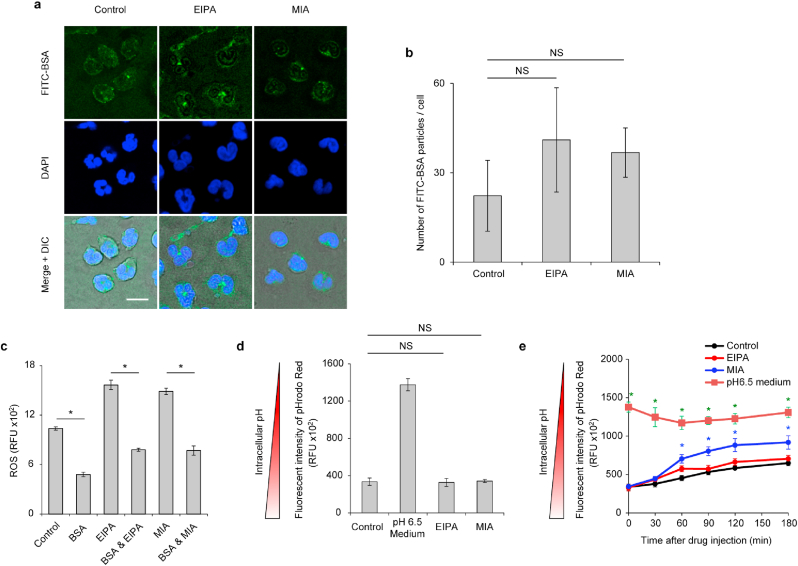
**| EIPA and MIA have no inhibitory effect on the uptake of serum albumin by neutrophils.** (a, b, c) Human neutrophils were cultured with the medium containing 75 μM of EIPA or MIA for 10 min (a, b), 2 h (c). (a, b) PBS solution of albumin-fluorescein isothiocyanate conjugate (FITC-Alb) was added to the medium at a final concentration of 0.02 g/dL. After fixation and staining with DAPI, cells were subjected to the confocal fluorescence microscopy analysis. (a) Representative images are shown. Bar = 10 μm. (b) Quantification of the number of FITC-Alb particles within neutrophils. (c) Intracellular ROS level in each treatment group was measured by DCFDA fluorescence. (d, e) Human neutrophils preloaded with pHrodo Red were incubated with the culture medium containing vehicle, 75 μM EIPA, 75 μM MIA, and pH 6.5 culture medium. (d) Intracellular pH represented by the fluorescent intensity of pHrodo Red 5-min after treatment. (e) Time-dependent changes in the intracellular pH. (b–e) Results represent the mean ± s.d. *P < 0.05 and NS = not significant by Dunnett's test (b, d, e) and Student's *t*-test (c). (e) The blue asterisks represent statistical significance between the control and MIA-treated groups. The green asterisks represent statistical significance between the control and pH 6.5 medium-incubation groups. (For interpretation of the references to colour in this figure legend, the reader is referred to the Web version of this article.)

Next, we tested the hypothesis that EIPA and MIA inhibit the uptake of serum albumin, which leads to the accumulation of intracellular ROS due to the lack of albumin-derived free thiol. We used a culture medium containing albumin-fluorescein isothiocyanate conjugate (FITC-Alb), which can be internalized and is microscopically detectable as small particles with green fluorescence [11]. Contrary to our expectation, the particles of FITC-Alb were still observed in the neutrophils treated with EIPA and MIA as well as the vehicle-treated control (Fig. 4a). No significant difference in the number of particles of FITC-Alb was detected among the neutrophils treated with vehicle, EIPA, and MIA (Fig. 4b). Moreover, replenishment of BSA to the EIPA- and MIA-containing culture medium prevented the accumulation of ROS (Fig. 4c), indicating that neutrophils even with the treatment of EIPA and MIA can internalize serum albumin and utilize it to degrade intracellular ROS. Since the decrease of intracellular pH is a key mechanism for the inhibition of macropinocytosis [16], we assessed the intracellular pH in the neutrophils treated with EIPA and MIA using the pH-sensitive probe pHrodo Red. The fluorometric analysis of pHrodo Red-loaded neutrophils showed that EIPA and MIA did not affect intracellular pH when EIPA- and MIA-induced intracellular Ca^2+^ increases occurred (Fig. 4d). In a longer observation period, a reduction was noted in intracellular pH in MIA-treated neutrophils; however, it did not reach pH 6.5 (Fig. 4e), which is reportedly sufficient to inhibit macropinocytosis [16]. Collectively, inhibition of the macropinocytosis of serum albumin is not the primary mechanism for EIPA- and MIA-mediated NETotic cell death.

### 3.5 A pharmacological approach targeting NCX inhibition-mediated signaling prevents EIPA- and MIA-induced NETotic cell death

**Fig. 5:**
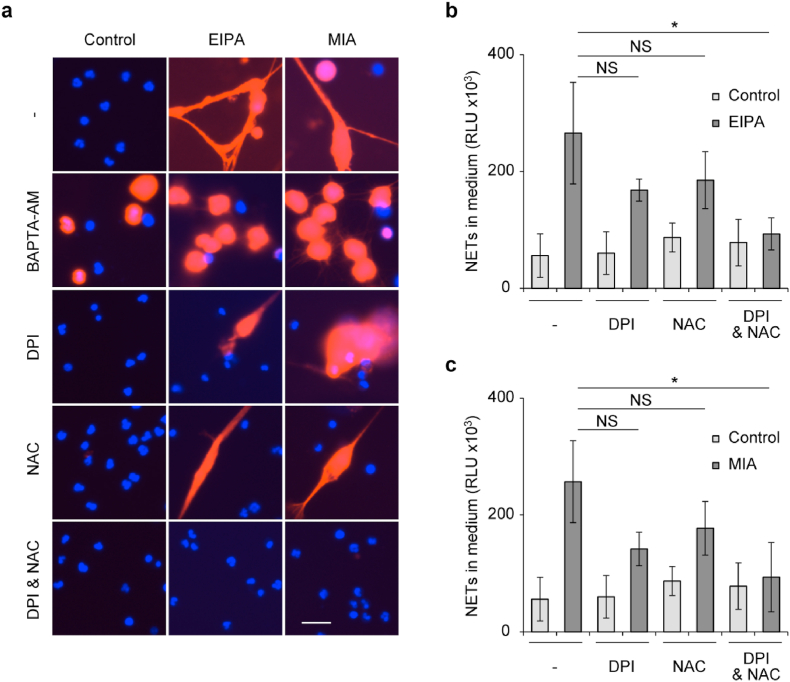
**| Inhibition of the NCX inhibition-mediated signaling prevents EIPA- and MIA-induced NETotic cell death.** (a–c) Human neutrophils were pretreated with 8 μM BAPTA-AM or 15 μM diphenyleneiodonium chloride (DPI) and/or 5 mM N-acetylcysteine (NAC) for 5 min, and then they were treated with 75 μM of EIPA or MIA for 5 h. (a) Representative images of neutrophils stained with Hoechst 33342 (blue) and SytoxOrange (red). Bar = 20 μm. (b, c) Levels of NETs within cell culture supernatant derived from neutrophils treated with EIPA (b) and MIA (c). Results represent the mean ± s.d. (*n* = 3; biological replicates, *P < 0.05 and NS = not significant by Dunnett's test). (For interpretation of the references to colour in this figure legend, the reader is referred to the Web version of this article.)

To confirm the involvement of NCX inhibition-mediated signaling (i.e., intracellular Ca^2+^ overload, NADPH oxidase activation, and intracellular ROS accumulation) in EIPA- and MIA-mediated NETotic cell death, we examined the efficacy of chelation of Ca^2+^ by BAPTA-AM, inhibition of NADPH oxidase by diphenyleneiodonium chloride (DPI), and degradation of intracellular ROS by NAC. Neutrophils treated with BAPTA-AM resulted in global cell death and its morphology was indistinguishable with or without the existence of EIPA and MIA (Fig. 5a and S7), suggesting that BAPTA-AM itself is cytotoxic to neutrophils. Since the concentration of BAPTA-AM used here was identical to the one required for the inhibition of intracellular Ca^2+^ overload (Fig. 2d) and NADPH oxidase (Fig. 2e), it is not reasonable to use a lower concentration of BAPTA-AM for reducing its cytotoxicity. Thus, BAPTA-AM showed sufficient inhibition against NADPH oxidase activation and intracellular ROS accumulation in EIPA- and MIA-treated neutrophils (Fig. 2e and f), but is not suitable for inhibiting the resultant NETotic cell death due to its cytotoxicity.

The single treatment of DPI and NAC modestly, but insignificantly, inhibited EIPA- and MIA-mediated NETotic cell death (Fig. 5a–c and S7). Then, we tested the efficacy of combined therapy of DPI and NAC on EIPA- and MIA-induced NETotic cell death. As shown in Fig. 5a–c and S7, concomitant treatment with DPI and NAC achieved the highest inhibitory effect against EIPA- and MIA-induced NETotic cell death. These results confirm our proposed mechanism that inhibition of Na^+^/Ca^2+^ exchange upregulates NADPH oxidase activity, elevates the intracellular ROS level, and triggers the resultant NETotic cell death, further supporting a model in which the forward mode activity of NCX act as a physiological regulator of intracellular Ca^2+^ balance and NET release.

## 4 Discussion

To date, little is known about infection-independent NET release. Tamoxifen, an estrogen receptor modulator [33]; microcrystals, such as MSU [26]; an excess of glucose [34]; autoantibodies against extracellular DNA and ribonucleoproteins [35]; and redox imbalance caused by albumin oxidation [11] are triggers of infection-independent NET release. In this study, we have discovered a new trigger of infection-independent NETotic cell death (i.e., inhibition of forward-mode Na^+^/Ca^2+^ exchange by amiloride analogs, EIPA and MIA). The reported IC_50_ values of EIPA and MIA for inhibiting NCX activity in rat pituitary cells are both approximately 130 μM [28], and, thus, the concentrations of EIPA and MIA employed in this manuscript were not high enough to induce non-specific cytotoxicity. We could not genetically demonstrate the involvement of NCX1 in NETotic cell death due to three experimental limitations: the short life of the isolated neutrophil does not allow us to perform either overexpression or knockdown of NCX1, the granulocyte-like cells differentiated from leukemic cell lines (e.g., HL-60) are not suitable for assessing the underlying mechanism of NET release due to the weak induction of NET release (data not shown), and the embryonic lethality of the NCX1 gene knockout [36] prevents us from examining the NCX1-deficient neutrophils obtained from the NCX1 knockout animals. However, our results presented here provide evidence that EIPA and MIA induce NETotic cell death by inhibiting forward-mode Na^+^/Ca^2+^ exchange and subsequently activating the downstream signaling.

In the present study, an increase in intracellular Ca^2+^ was observed in EIPA- and MIA-treated neutrophils, but not in those treated with other amiloride analogs that reportedly function to inhibit NCX activity [12,13]. We speculate the reason for this difference between the previous study and ours may be due to differences in the experimental samples (porcine or bovine cardiac sarcolemmal plasma membrane vesicles *vs.* human neutrophils), incubation media (MOPS-Tris buffer containing NaCl, CaCl_2_, and KCl *vs.* RPMI-1640 medium containing 1% FBS), and methods for the binding of amiloride analogs to NCX (voltexing of the reaction mixture *vs.* simple incubation). Regarding the difference in the inhibitory effects on neutrophil NCX among EIPA and MIA *vs*. other amiloride compounds, we propose the following reason: the longer alkyl group on the 5-amino nitrogen atom in EIPA and MIA contributes to the hydrophobic interaction with Na^+^ binding sites in NCX and the resultant disruption of Na^+^ binding to NCX, which is not induced by other amiloride compounds (Fig. S4).

Mitochondria also possesses a Na^+^/Ca^2+^ exchanger (NCLX) that produces net Ca^2+^ efflux and Na^+^ influx [37]. To the best of our knowledge, there have been no studies to show whether the activity of NCLX influences NET release or if amiloride analogs affect the activity of NCLX. However, it is theoretically possible that the conditions that evoke the overactivation of NCLX (e.g., intracellular Na^+^ overload) may contribute to cytosolic Ca^2+^ overload and resultant NET release. Future studies are needed to elucidate the potential role of NCLX in cytosolic calcium-triggered NET release.

Amiloride and its analogs are also known to cause inhibition of macropinocytosis, which hypothetically leads to the intracellular starvation of serum albumin, a resultant increase of intracellular ROS, and NET release. However, even EIPA and MIA, both of which efficiently triggered NET release, did not inhibit the uptake of serum albumin by neutrophils and, moreover, did not induce the sufficient decline of intracellular pH (Fig. 4d) that drives amiloride-induced macropinocytosis [16]. Therefore, we propose the following mechanism to explain the ineffectiveness of EIPA and MIA in the inhibition of albumin uptake: EIPA- and MIA-induced inhibition of NCX occurs rapidly (Fig. 2b) and thus there is not enough time before the effect of NHE inhibition is manifested. Neutrophils can internalize serum albumin by endocytosis [38] as well as macropinocytosis. Furthermore, the induction of macropinocytosis requires specific manipulations, such as starvation of serum [16] or glutamine [39], or the addition of epidermal growth factor [40] or macrophage colony-stimulating factor [41]. This suggests that macropinocytosis is not constitutively active and other internalization systems, such as endocytosis, are alternatively active in the non-starved and physiological condition.

Our findings also have significant implications for future clinical investigations. As a number of studies demonstrated the anti-tumor activity of amiloride and its analogs, including EIPA and MIA, against various human cancer cells [39,[42], [43], [44], [45]], it is likely that clinical trials to evaluate their efficacy will be conducted. Thus, it is necessary to inform the clinical trials examining the anti-tumor effect of EIPA or MIA of the potent risk of NET-mediated thrombosis and cancer metastasis. To our knowledge, there are no published studies which aim to broadly identify potent inducers of NET release by drug screening approaches. Cancer patients often develop chronic diseases, such as hypertension, heart disease, and diabetes [46], and take medication for them; therefore, future work focusing on identifying thus far unknown inducers of NET release may further contribute to the prevention of thrombosis and cancer metastasis by replacing drugs that induce NET release.

Overall, our study shows that inhibition of Na^+^/Ca^2+^ exchange by EIPA and MIA triggers infection-independent NETotic cell death. Our findings identify a previously unknown mechanism in NET release and provide further insight to the role of the forward mode activity of NCX in the regulation of intracellular calcium balance and NET release.

## Declaration of competing interest

The authors declare that they have no known competing financial interests or personal relationships that could have appeared to influence the work reported in this paper.
