# Supplementary material for: Pharmacological inhibition of sodium-calcium exchange activates NADPH oxidase and induces infection-independent NETotic cell death
Source: Redox Biol. 2021 Apr 26;43:101983. doi: 10.1016/j.redox.2021.101983 (PMC8105669; doi:10.1016/j.redox.2021.101983)
Supplement: Multimedia component 9 [file mmc9.docx]

Supplementary Materials for

**Pharmacological inhibition of sodium-calcium exchange activates NADPH oxidase and induces infection-independent NETotic cell death**

Minoru Inoue^#1^, Masahiro Enomoto^2^, Michio Yoshimura^1^, Takashi Mizowaki^1^

# **Address correspondence to:**

Minoru Inoue, MD PhD

Email: miinoue@kuhp.kyoto-u.ac.jp

**This file includes:**

Table S1

Figs. S1 to S7

The legends for Video S1 to S4

**Table S1. Amiloride and its analogs examined in this study.**

|  | Structure | Molecular weight |
| --- | --- | --- |
| Amiloride |  | 229.63 |
| EIPA |  | 299.76 |
| MIA |  | 299.76 |
| DMA |  | 257.68 |
| Phenamil |  | 305.72 |
| Benzamil |  | 319.75 |

The 5-amino group in each molecular structure is highlighted in red.

EIPA: 5-(N-ethyl-N-isopropyl)amiloride, MIA: 5-(N-Methyl-N-isobutyl)amiloride, DMA: 5-(N,N-Dimethyl)amiloride

**
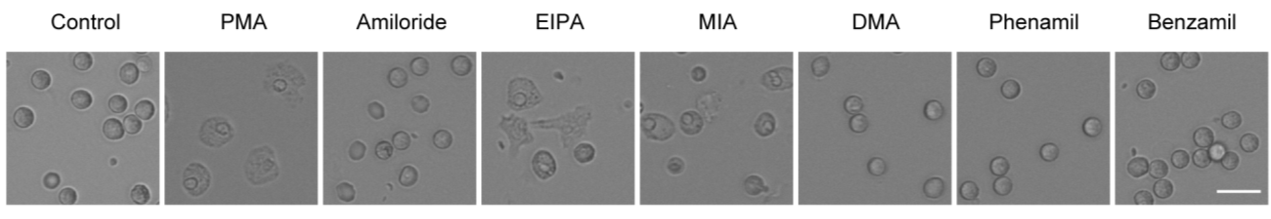
**

**Figure S1 | Morphological change in neutrophils treated with amiloride and its analogs.** Human neutrophils were treated with the indicated compounds (final concentration: 75 μM for all amiloride compounds and 20 nM for PMA) for 2 hours. Bar = 20 μm.


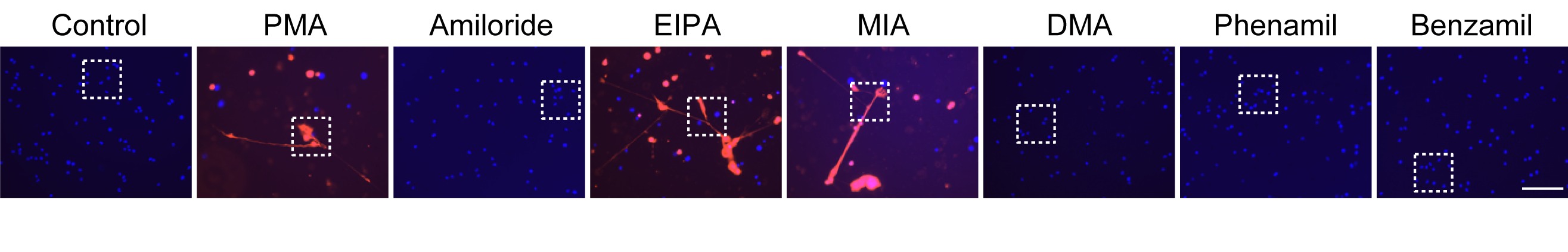


**Figure S2 | The effect of amiloride and its derivatives on human neutrophils.**　Human neutrophils were treated with the indicated compounds (final concentration: 75 μM for all amiloride compounds and 20 nM for PMA) for 5 hours. Subsequently, neutrophils were stained with cell-permeable DNA dye, Hoechst 33342 (blue), and cell-impermeable DNA dye, SytoxOrange (red). Representative images are shown. The view in the dotted square is enlarged in Figure 1a. Bar = 100 μm.


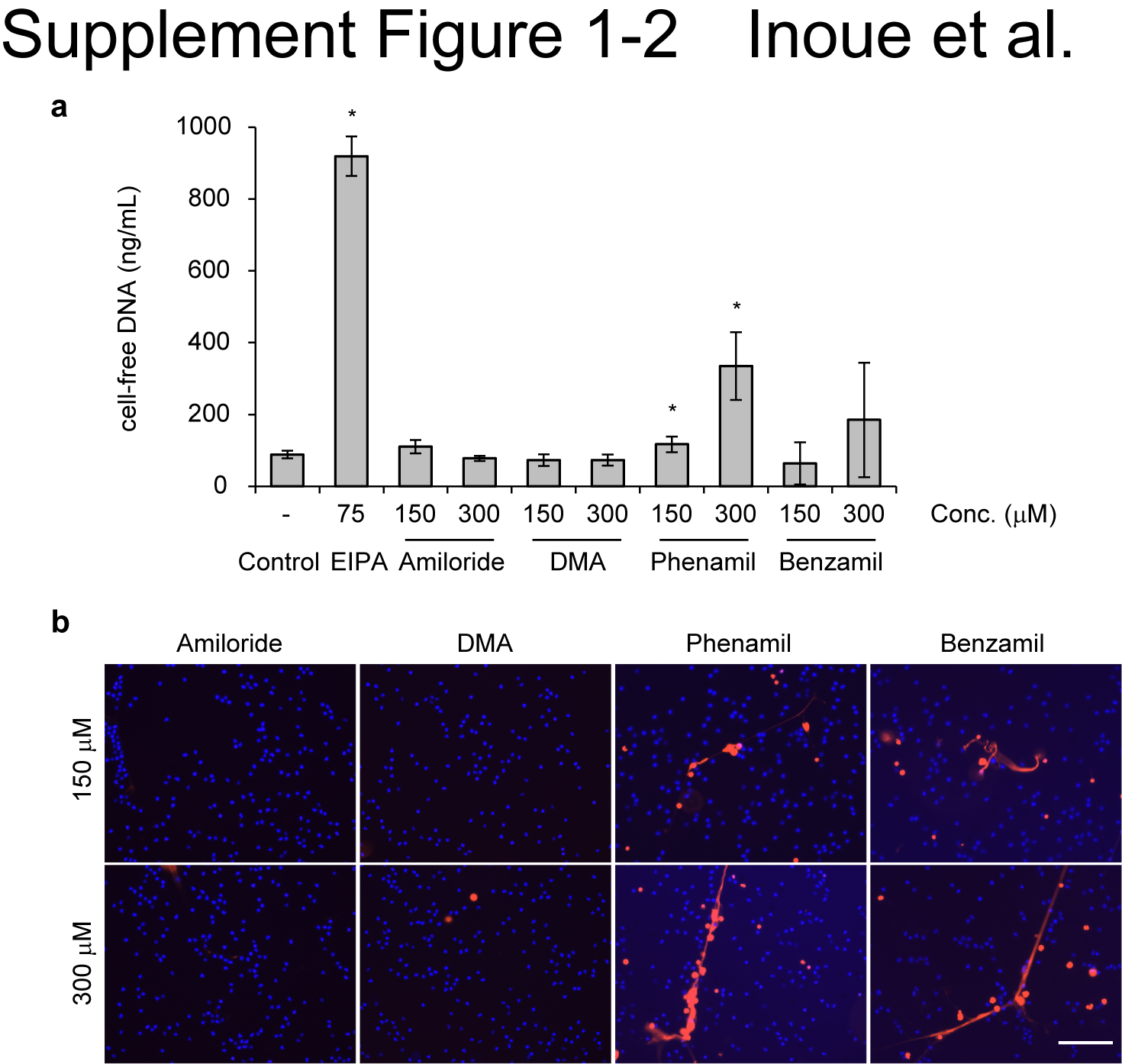


**Figure S3 | The effect of higher concentrations of amiloride, DMA, phenamil, and benzamil on human neutrophils.**　(a, b) Human neutrophils were treated with 150 μM and 300 μM of amiloride, DMA, phenamil, and benzamil and its analogs for 9 hours. (a) Concentrations of extracellular DNA within culture medium. (b) Neutrophils were stained with cell-permeable DNA dye, Hoechst 33342 (blue), and cell-impermeable DNA dye, SytoxOrange (red). Representative images are shown. Bar = 100 μm. Results represent individual values with the mean ± s.d. (*n* = 3; biological replicates, significant differences were compared with the control at **P*<0.05 by Dunnett’s test).


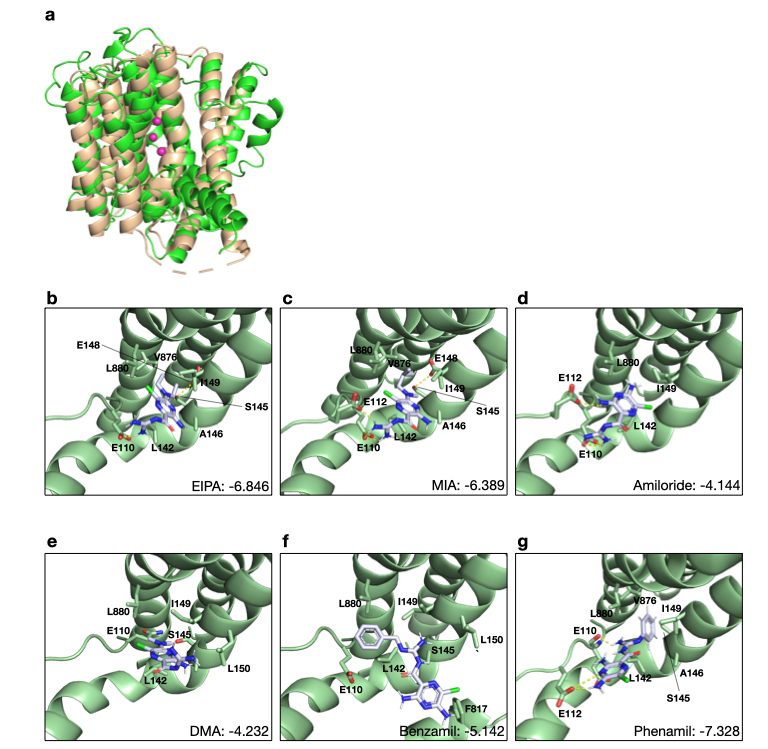


**Figure S4 | Computational docking models for amiloride and its derivatives.**(a) A homology model of human NCX1 (green) superimposed onto *Methanocaldococcus jannaschii* NCX x-ray crystal structure (wheat) (PDB 3V5U). Magenta spheres represent Na^+^. (b-g) For each ligand, a docking model in which the ligand interacts with the transmembrane helices constituting the Na^+^ binding sites was chosen from the three lowest-interface_delta-energy structures. The number shown in each panel represents interface-delta energy, which is calculated by subtracting the total energy with the ligand unbound from that with the ligand bound. Yellow dotted lines indicate hydrogen bonds. Residues which interact with amiloride or its derivatives though hydrogen bond or hydrophobic interaction are labeled with residue numbers. Only helices and loops containing the residues that interact with amiloride or its derivatives are visualized for sake of simplicity.


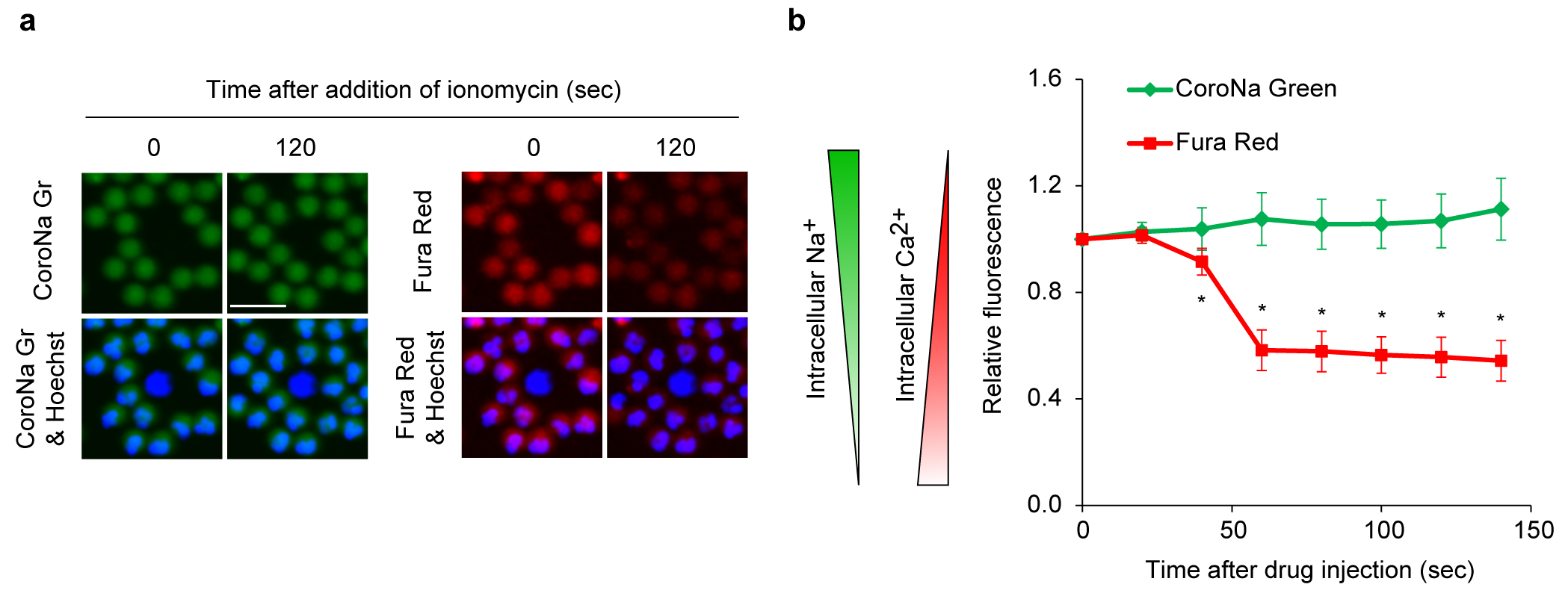


**Figure S5 | Ionomycin promotes Ca^2+^ influx without simultaneous Na^+^ efflux.**　Human neutrophils loaded with Fura Red AM and CoroNa Green AM were subjected to the time-lapse imaging. One micro-molar of ionomycin was added to the culture medium at time 0. (b) Representative images are shown. Bar = 20 μm. (c, d) Changes in the intracellular Ca^2+^ and Na^+^ after administration of ionomycin. Intracellular Ca^2+^ and Na^+^ were analyzed using the fluorescent intensity of Fura Red AM and CoroNa Green AM, respectively. Each fluorescent intensity was normalized by the one measured at time 0. Results represent the mean ± s.d. (*n* = 20 cells, **P*<0.05 by Student’s *t*-test).


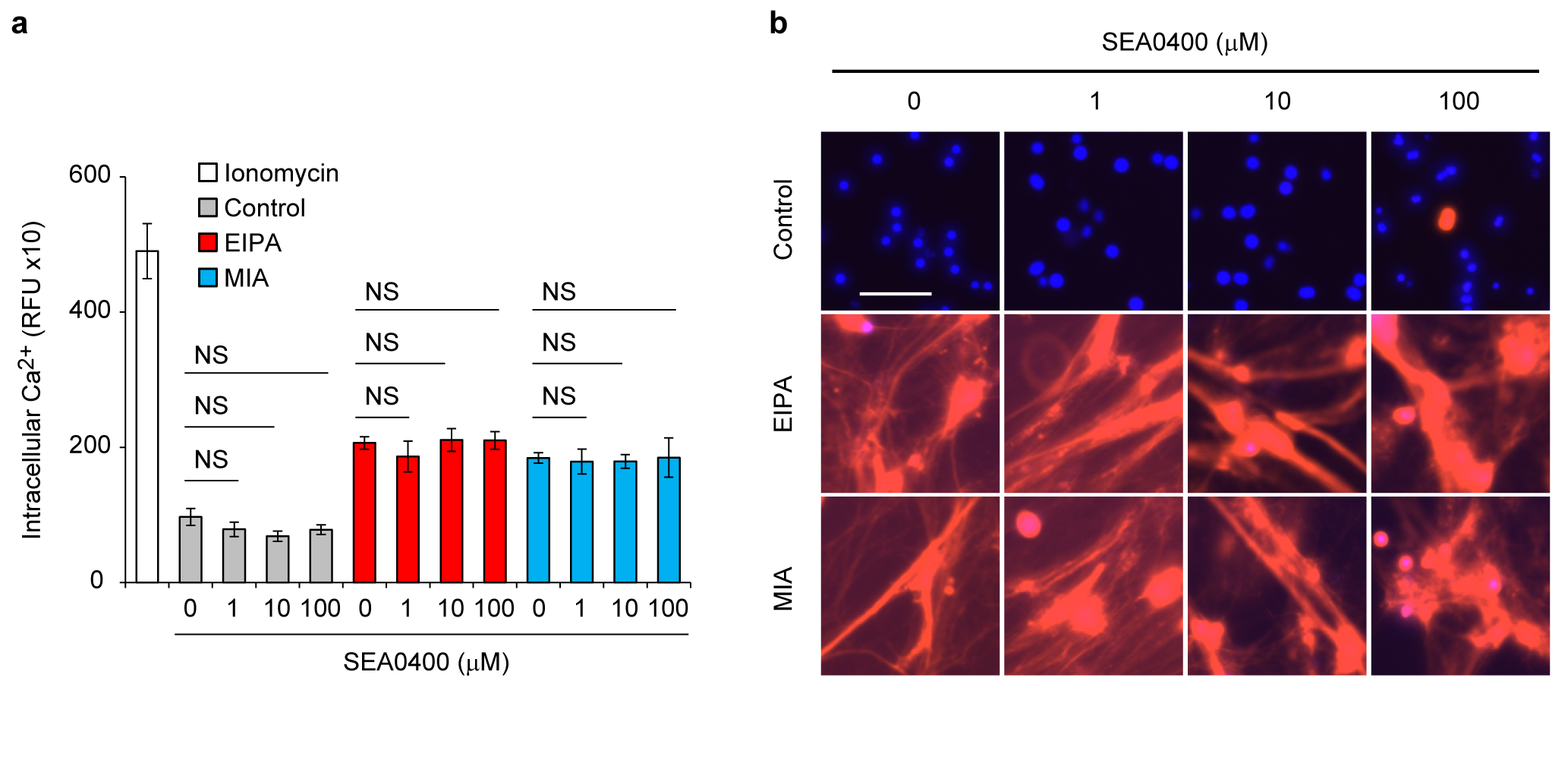


**Figure S6 | Effect of the reverse-mode NCX inhibitor SEA0400 on neutrophils with or without the EIPA or MIA treatment.**　 Human neutrophils were treated with SEA0400 (final concentration: 1 μM, 10 μM, and 100 μM) with or without 75 μM EIPA or 75 μM MIA for 5 min (a) and 5 hours (b). (a) The fluorescent intensity of Fluo-4 AM was measured. Results represent the mean ± s.d. (*n* = 3; biological replicates; NS = not significant by Dunnett’s test). (b) Representative images of neutrophils stained with cell-permeable DNA dye, Hoechst 33342 (blue), and cell-impermeable DNA dye, SytoxOrange (red). Bar = 50 μm.


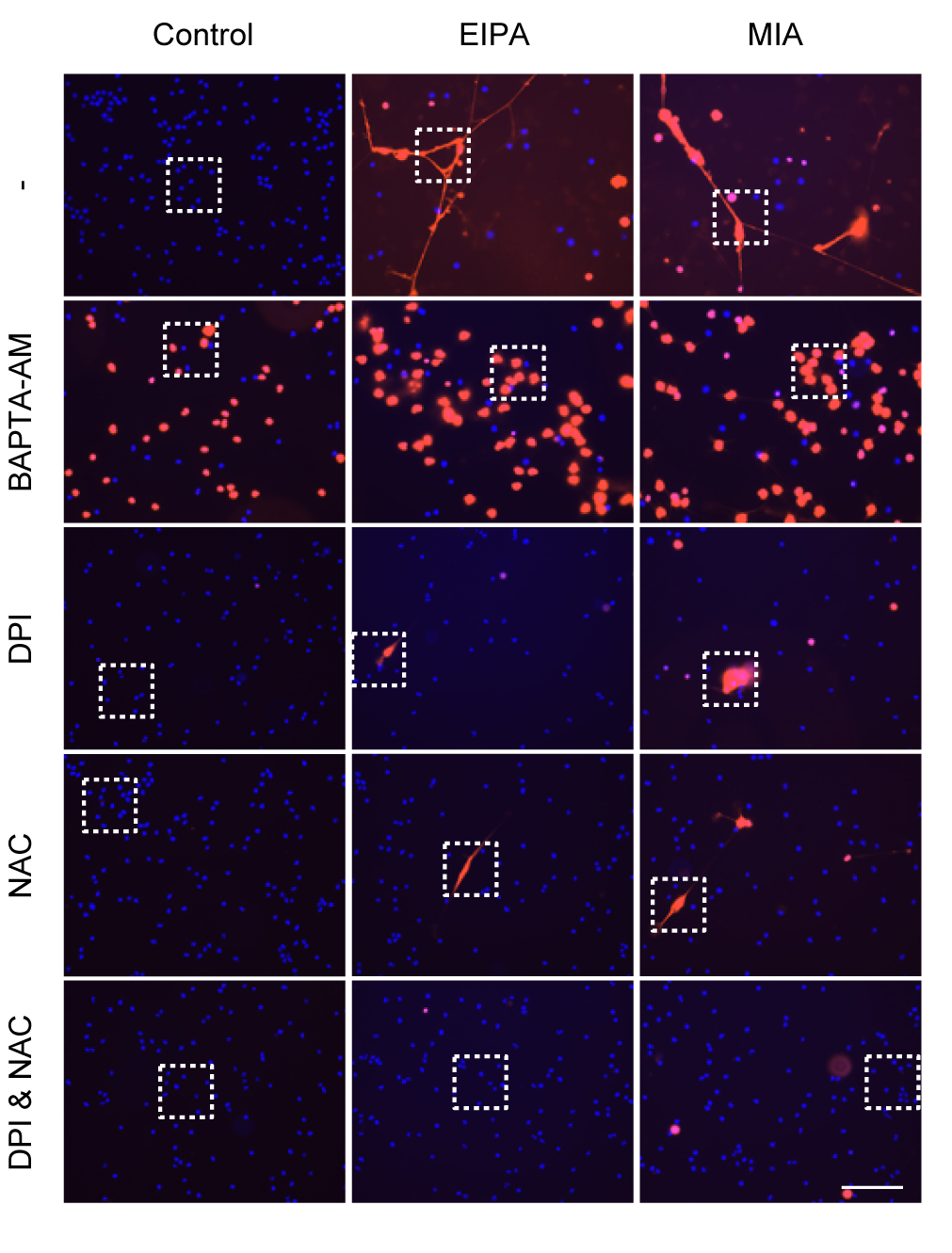


**Figure S7 | The effect of** **BAPTA-AM, DPI, and NAC on human neutrophils with or without the existence of EIPA and MIA.**　Human neutrophils were pretreated with 8 μM BAPTA-AM or 15 μM diphenyleneiodonium chloride (DPI) and/or 5 mM N-acetylcysteine (NAC) for 5 min, and then they were treated with 75 μM of EIPA or MIA for 5 hours. (a) Representative images of neutrophils stained with Hoechst 33342 (blue) and SytoxOrange (red). Bar = 100 μm. The view in the dotted square is enlarged in Figure 5a. Bar = 100 μm.

**Video S1 | Time-lapse imaging of intracellular Ca^2+^ and Na^+^ in EIPA-treated neutrophils.** Human neutrophils loaded with a Ca^2+^ indicator Fura Red AM and a Na^+^ indicator CoroNa Green AM were subjected to the time-lapse imaging. One micro liter of 75-mM EIPA dissolved in 100 μL of culture medium was added at time 0 (final concentration of EIPA: 75 μM). The intracellular levels of Ca^2+^ (a) and Na^+^ (b) were shown in red and green, respectively. The increase in Fura Red AM fluorescence corresponds to the decrease in Ca^2+^ concentration. The increase in CoroNa Green AM fluorescence corresponds to the increase in Na^+^ concentration. The cell nuclei were stained with Hoechst 33342 (blue). Time in min:s.

**Video S2 | Time-lapse imaging of intracellular Ca^2+^ and Na^+^ in MIA-treated neutrophils.** Human neutrophils loaded with a Ca^2+^ indicator Fura Red AM and a Na^+^ indicator CoroNa Green AM were subjected to the time-lapse imaging. One micro liter of 75-mM MIA dissolved in 100 μL of culture medium was added at time 0 (final concentration of MIA: 75 μM). The intracellular levels of Ca^2+^ (a) and Na^+^ (b) were shown in red and green, respectively. The increase in Fura Red AM fluorescence corresponds to the decrease in Ca^2+^ concentration. The increase in CoroNa Green AM fluorescence corresponds to the increase in Na^+^ concentration. The cell nuclei were stained with Hoechst 33342 (blue). Time in min:s.

**Video S3 | Time-lapse imaging of intracellular Ca^2+^ and Na^+^ in vehicle-treated neutrophils.** Human neutrophils loaded with a Ca^2+^ indicator Fura Red AM and a Na^+^ indicator CoroNa Green AM were subjected to the time-lapse imaging. One micro liter of DMSO dissolved in 100 μL of culture medium was added at time 0. The intracellular levels of Ca^2+^ (a) and Na^+^ (b) were shown in red and green, respectively. The cell nuclei were stained with Hoechst 33342 (blue). Time in min:s.

**Video S4 | Time-lapse imaging of intracellular Ca^2+^ and Na^+^ in ionomycin-treated neutrophils.** Human neutrophils loaded with a Ca^2+^ indicator Fura Red AM and a Na^+^ indicator CoroNa Green AM were subjected to the time-lapse imaging. One micro liter of 1-mM ionomycin dissolved in 100 μL of culture medium was added at time 0 (final concentration of ionomycin: 1 μM). The intracellular levels of Ca^2+^ (a) and Na^+^ (b) were shown in red and green, respectively. The increase in Fura Red AM fluorescence corresponds to the decrease in Ca^2+^ concentration. The increase in CoroNa Green AM fluorescence corresponds to the increase in Na^+^ concentration. The cell nuclei were stained with Hoechst 33342 (blue). Time in min:s.
